# Supplementary material for: Satellitome Analysis on Talpa aquitania Genome and Inferences about the satDNAs Evolution on Some Talpidae
Source: Genes (Basel). 2022 Dec 31;14(1):117. doi: 10.3390/genes14010117 (PMC9859602; doi:10.3390/genes14010117)
Supplement: Supplementary file 1 [file genes-14-00117-s001.zip › Supplementary Figures.pdf]

Supplementary Figures

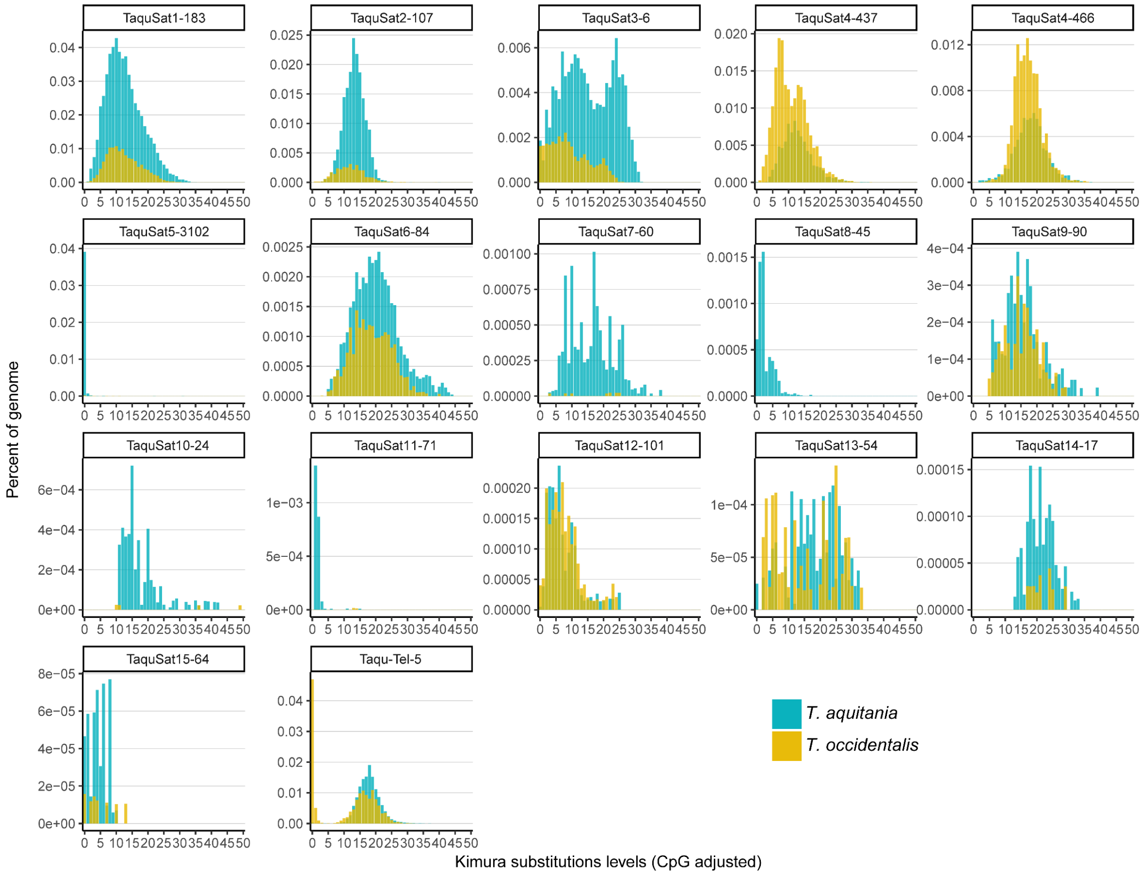

Figure S1. Satellitome landscapes (abundance versus divergence) of *Talpa aquitania* satDNA families on *Talpa aquitania* and *Talpa occidentalis* genomes.

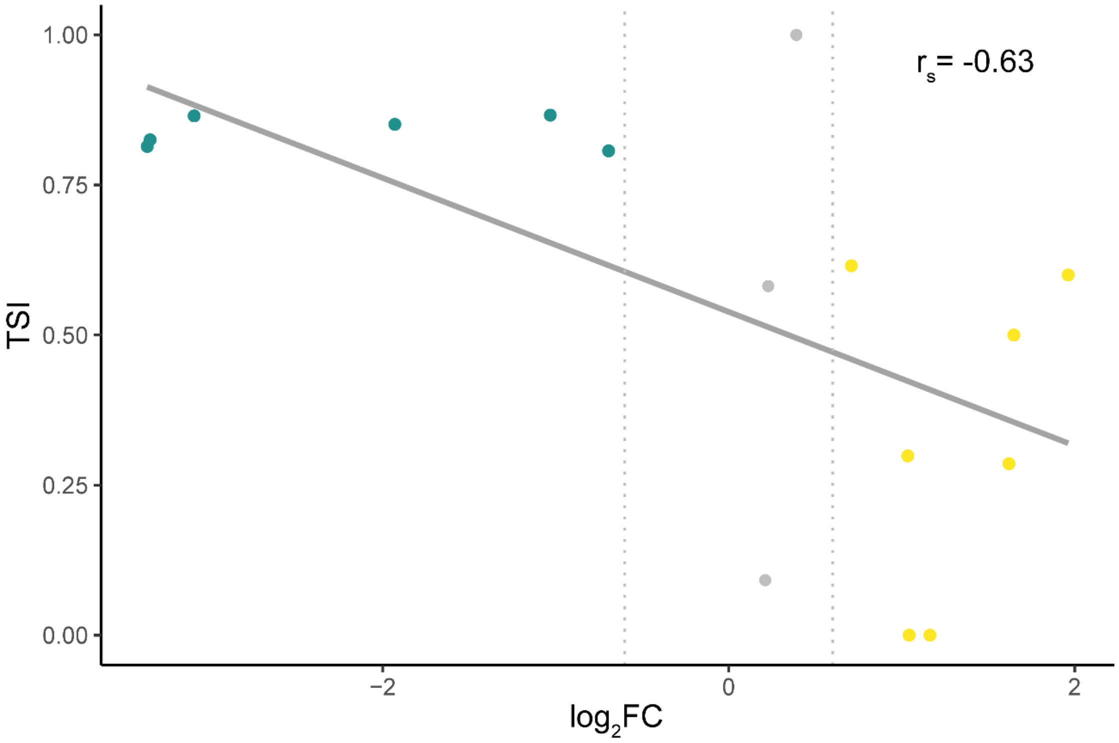

Figure S2. Correlation between the tandem structure index and the fold-change of satDNA family abundance among *Talpa occidentalis* assembled genome and *T. occidentalis* genome. The fold-change is expressed as log2 of genome percentage among samples. SatDNA families with less of 1.5 fold-change between samples (-0.6 < log2 ratio < 0.6) are in grey, most abundant satDNA families on T. occidentalis assembly in yellow and most abundant on *T. occidentalis* unassembled reads in blue. Regression line is depicted in dark grey.

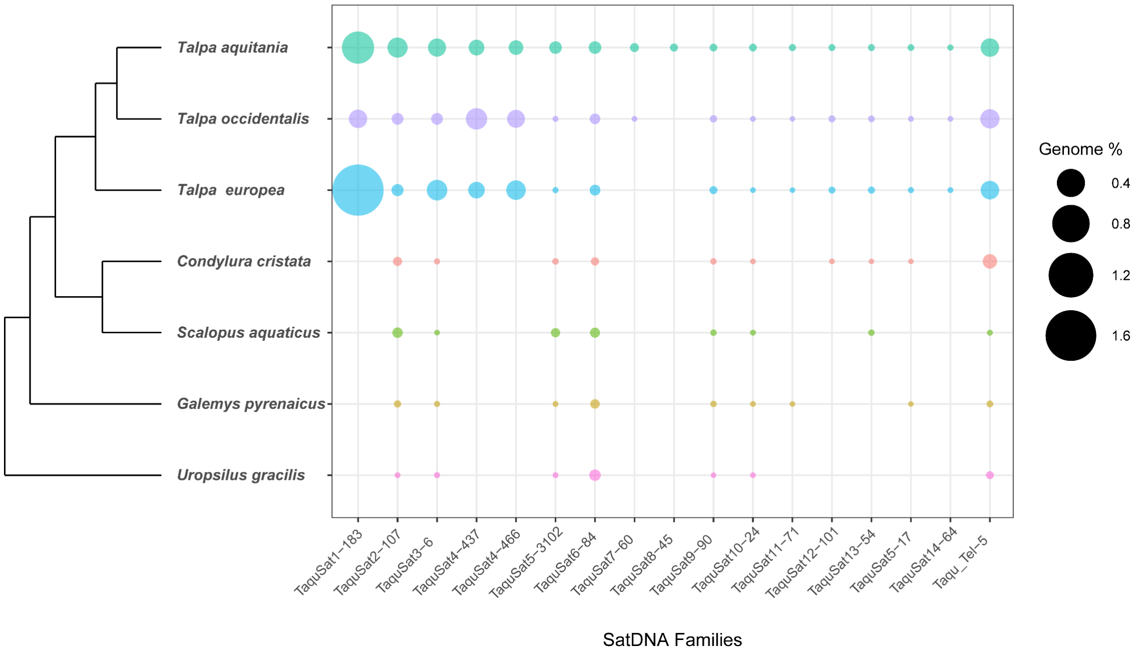

Figure S3: Presence and abundance of *Talpa aquitania* satDNA families across species of the family Talpaidea. The bubble size indicates the abundance of satDNA family.
