# Supplementary material for: Satellitome Analysis on Talpa aquitania Genome and Inferences about the satDNAs Evolution on Some Talpidae
Source: Genes (Basel). 2022 Dec 31;14(1):117. doi: 10.3390/genes14010117 (PMC9859602; doi:10.3390/genes14010117)
Supplement: Supplementary file 1 [file genes-14-00117-s001.zip › Supplementary Tables S1 and S2.pdf]

Table S1: *T. aquitania* satDNA families consensus sequences

satDNA name	consensus sequence
**TaquSat1-183**	GAGATGTAGAACTACAAGAACAACAACAAGAACTAGAAGAACAAGAAGAACAAGAAGAAGAAACAGAAGAATAAGTCAAAAAA<br>GCAGAAATAACAGAAGAATAAGCTGAGGAAGAAGCAGAAAAAGAAGTAATTGAAAAAGCAGAAAATGCAGAAGAAACAGAAGA<br>AGATGAAGAAGAAGAAG
**TaquSat2-107**	TTTCTCCTCAGAGGCCTGTCTTCTCCTCAGAATCTGCTTCCCAGGGTGTCATTTCTCCTCAGATATTTCTCCTCAACACTCCT<br>TACTACAGTATTTCTCCTCAGTTT
**TaquSat3-6**	CTTCTT
**TaquSat4-437-470**	Consensus pattern 437 bp<br>GGTGTGCCCCGAGGGGAAGATTTACCGTTCCCCCAAGGGGGAAGAGGGTCGCGTTTCCCCCTGGGGTAGAGGGTCCCCTACCC<br>CCGTGGGGGCTGAGGGTCCCCATCCCCCTCGCCGGCGCCCCCTCGCGGCGGCTCTGTGAAGTGCACCTGTGCTCAGGGAGAGT<br>CCGGTGGGCGGGGTCAGGCTCCGGCCGGGGGCGGAGCCTGGGGCTCATCAGCGCTGCCTGGCCAGGTGAGGACTCTTCCCCTT<br>TGAGTGGTGCTGGGTTGCTCGGGCCTTTCCCCGCCGGCCTGTCTTCCTGCAGTTTTGGAGGAGGCCGCGGTCGCCGTCTGTCT<br>GTGGCTTGCAGGGGGTGCCCTCCCTGACGGTGACCCCGCCCCTTCGTCTATGGCGTCTTGGGCGCAATGTCCGCCCCTCGGGT<br>TGTGAGTGCGGAGGAGGTTGCA<br>Consensus pattern 466 bp<br>GGCTTCCCCCAAGGGGAAGATAGCCCCGTTCCCCCATGGGGGAGGAAAATCCCGTTCCCCCATGGGTGCAGAGGTCCCCCAAA<br>CCCCATGAAGGCTGAGGTCCCCGAACCCCCTCACCAGCGCCCCCTAGCGGCAGCCCTGAGAAGTGCACCTTCGAGCATGCAGA<br>GTCCAGTGGGCGGGGTCAGGCTACGGCCAGGGGCGGAGCCTGGGGCTCATCAGCGCTGCGGGCCAGGTGAGGACTCTTACCCT<br>TTCAGTGGGGCTGGCTTGCCCGGGGCTTTCCGCGCCACCCTGTCTGCCTGCAGTTTTGGAAGAGGCTCAGGTCGCCGCTTGTC<br>CCTGGCGTACAGGGGGTGCCCTCCCCGGACAGGGACCCCACCCCTCCGTCTAGGGCGCCTGAGGGCGCATCGGCATGCCCCTA<br>GGGCTGTGAGTGCGAGAGGAGGGTGCAGGTGTTCCCCGGGGGAAAGATTTG
**TaquSat5-3102**	TGATCATAGATGGGGAAATACCAATCCACATCAACAAGAAGAGGGGGTTCCTCTTCCTGAGGACACAAGTGGATTATTCCACC<br>TTTCAGGCTCTACATGAACTGCCTTCCATAGGAGAGCTGGCGTAATATAAATATTTATGAAAAGAAATGCACTAAAACTAAGA<br>TGAAAGTGGGCGCAGACCCAGAGAGCCACAGGGTGGGGGGTACCTAGAAGTCAGTGACCTGGCACCCCTTTGAAACAACCATT<br>TGCTTAACAGGGACTTGGGATTTGCCAGACAAGATATCGATTGCACAGAAGCAAGAAGGACTTGGTTTGGTTTGGAGGCAGGA<br>ATGAAGCATGACAATGAGCATCACATTGTGCTCCTTACCTCTCCCTGGAGGGTTCTGCCTCCCAGGTTCTGCAGCGGCTGCTT<br>TCTCCTCCACATTTTCCACGATCTCTAATCTTGAAGATAGAACAAACTGGTCATCCATACAAATGACCAAACCAAGTGCTTTA<br>CTAGACCTGAAACTGTAGTTGAGGACCTCAACTCCACCCTGAAGCAGAATCACCACAGCCTGCTACCTGCTTGGCTAGTGGGC<br>GGCTTCCCTGCGCTGCTCCTGCATCTCCACTCCATCTGACACGCCAGGACCTGTGGGGAAACACAGCCACTGGTCAGGCCCAG<br>TACAGTGCAGAGAAGGGGGGAAGGAGAGTCAACTTACCCATTTCTCCTGAGCCTTCCTTCCAGCCTGATCCACCGAAACTGAT<br>CCTTGGAGCCTGAGCCTTTGAGCCTTCAAGCCCTGGAGCCTTCTTCTCCTGCTGGGCAATCTGAAAAGAGCAACACAGCCAAG<br>CCCTGGGGTTAGGTCCCTGGGGCTTTGCAAACATTTGTTTGAGGAGCAGGAGGATTGTCTGTGGGGAGAGGCAGGTTACCTGG<br>AGTTGATGAACAAAGTCAGCTTGCCTCAGTTTTTCTGCAGTTTCTTCAGTTTCTTGCCTATTATGTTTGATTTCATTAGAGAA<br>AGCAGAATATTAAGAATCATTTAAAGTCATTTCCCAAGTGCCTATTACCTTCACATCTATTGTTCCCTAATACAACTGACCTA<br>TACACAGAAAACAATTTTTCAAGGTTATAAACACACTGTGGGGTCAAAGCTTTTTGATGCATCATTTCCTTATTTACTGTTTG<br>ATGCCCACCAGATTCTCAGCCTGTGTAACTTACTTACCCATCTCAACATTTCGCTAATTGATCATGTCCTCAATAGCTTGTCT<br>CATAAAGCTCTAATCTCCTTTGATTTATATCCCTATCATATTTGCCAGAATTTCGAAAGTCTAGGTCCATCAAATGACAAAAG<br>CATATTTACGGATATGATTCACCCTACAGTGAGCTAGTTTGTTCGTGATTATAAAAATTACACTGTCCTCAGAGACATTTGTA<br>TGAACTAAAAGGCTAAGAGCATTGGGGGATCAAGAAATATTTATGAACTTATTTTTTGTTGTTCTTTGTAGAACAACTTTTGT<br>TCCTTGTGTTGTGTCTCTATATGACTTCTGAAACTTTGCTGATATCACTAAACATCCTGAGTCTAAACATTACAAGTTTAAAA<br>ACAAAAAGCCGGGATTTCTTGACCTGCTGGTATCTACGAACTCACTTGTATTTTTCAATTTCTTGAAGAATTTCTTTCAGTTT<br>CATCTCCAGAGCTACAAGCTCCTTCTCCTTTTTCTCCCTTTCATGCTGTTTCCATTTCAGCATCCTGAATTGGGAAAGACAGA<br>GATGATGCCCAGCACCCCTCACGAAATCCTGCCTTTCCCAGCTCAGGGCCTGGATTGCAAAGCCCCCTCCTGCGCTGGCCTCT<br>GCTGTGCCCTCTGCAAACTCGGCTTTTCCTCACAGTCTCCACCAGCTCATTGCTCTTCAGTCATCAGGACAAGGGCGTGGGGT<br>GCCTCCCTGTGCTGGGGCTGCTGTTTGAGAAGGTGAAGGCTTTTTCTTTCAAACTGGAAACGTACACGTGAAATTTCCACCTT<br>GAAATAAAGGGGATGCCTGTCCAACTCACGACAACCACATACATTAGGGCAAAGAAGGGGATCCTACCAGTCCGCATCTCCGA<br>GGGGAGCCTGATTATCAAGCCCAGGCTGTGCTTATGCAGGACAAGTAGCTATGAAGACAGTCTTACAGGCTGGGACTTCCCGA<br>GCCCTCTGCCATCAGCCCCACATAGTCTGAAGGCTCGGGAGCAGAGCTTTGCCTCCTACTGCAAACACCTCGTGGGAGAAAAG<br>GTCCATCTTATCGCTGCTAGGATGTACTGAAATATAAAATGCTGGAAACTGTCCCTTAGTCAGGGTATTATCTCTAGGAAGCA<br>ATTTCAAATGGACAACAAACAGCTTCAGGGCGTCAATTTCCTTTACCAAGACCCAGATCACAGCTAGTGTGGGCAGCAATGAG<br>CTGTGGCTGGGAATCCTTCCTTTGCTCTGCCGCCCTCAGACTATTTTTCTCATGCTGCTGACTCCAAGCTGACACTCTATTAA

	CTGTATAGATTGACACACAAAATAATAAGGAACCGGGACAAAGTAAGCACTGAAGAGAGAAGAGGACACACACTTCTTTCTAGCCTCCCTACTGTTCCTGCAGCAACCGCTTCCTATAGTGACTGCAGTACTTATGGCTTTGCTGTTTACATGCCCACATTACATGCCCACAGAAAAAGATCACAGAGGAAGGAAAGCTGGTGGGGACATCATCCCCCTACCCTTCTCTGCGTCCTCAGCTGGCACAGAGTTGCCATCACTCACGGGAGCTGCACCAGGTCCTGAGTCTGACAAAGACCACAGAAGTGACAGAGGTGTATTGGGACTTGCAGAGATGGTGAGGAAACCCTCCTTGCCTTCCCCCATTACCCTCCATTGTCCCCACATTCAGTCCCTTCTCAGGATATGGGGCTCTCCAGGAGCCTCACTGGCCCCAGAGCCCAAGGGGGACAGGAATCACCCCAGTCCCCTGGGTCAGACGCTTTCTCTCAACTTCATCACCCAAGTGGGAACCTGTCAGGGCTCACCTAGTTGGAGTCTGGCACAAAAACAGAGGCTCCCCAATGCTTGGCAAGACAAATTGCCATTTCAATGTAGGTTAAAGAAAAAGTA
**TaquSat6-84**	GAAACCYTAYRAATGTGAAGARTGTGGGAAAGCYTTTWMCCRKAAGTCAHMCCTCACTRAACATCAGAGAATTCAYACTGGAGA
**TaquSat7-60**	GATCTGTCAGCAGCTAGGTATGCCTTGCGATCACAAGCCTCAACTAGTGGTACCTGGGTT
**TaquSat8-45**	GGCTGAAGACCATCCACCAGTCTCATCAACATCTCGAGACGTAAA
**TaquSat9-90**	GAACCAAGTACAGAGGANAGGAGACAAATGATAGCACAGGGTGGAGGCTCAGATGAAGANAGGGAATACAGACAGRSTAGAAGGAATCTA
**TaquSat10-24**	GAGCCTTGATCATGGTCACAGACT
**TaquSat11-71**	TCAGCCTACCCCCCAAGGGGTCTACAGCCACTCACAGTTTAACAGGCAGTGCCTCAGCGCCTGCCACTGAAG
**TaquSat12-101**	CGGAGGAAAACCACTGAATGACAGGACTTGGTGTTGAGAGCCTGGAAAGTGACCTGCGTGGAGTCGGGCTTCTACATCTCCACAGTCTGAGAATAACAAAA
**TaquSat13-54**	CATGCAGGATCAAGTGCAACGACAAAATCAGACTTTTGAGTCAGCTTTTCAAAG
**TaquSat14-17**	GACCCCAGAACCCCAGT
**TaquSat15-64**	AGCCTGCCGCAGAGCAGGCTGGGAAGGATGAGACCAAGCAATAGAATAAAGAGGGCTGCCTTCA

Table S2: List of oligos used to localize by FISH the repeat DNA sequences of *Talpa aquitania*

Oligo	Sequentia (5' a 3')
TaquSat1-183-F	AATAAGTCAAAAAAGCAGAAATAACAGAAG
TaquSat1-183-R	TTGTTCTTCTAGTTCTTGTTGTTGTTC
TaquSat2-107	TCWCCTCAGATWTTTCTCCTC
TaquSat3-6	TTCTTCTTCTTCTTCTTCTTCTTCTTCTTC
TaquSat4-437-466-F	TGCCTGCAGTTTTGGAGG
TaquSat4-437-466-R	GACTCTGCCTGCGCACAG
TaquSat5-3102-F1	CACATCAACAAGAAGAGGGGGTTC
TaquSat5-3102-R1	TGAGTTCGTAGATACCAGCAGGTC
TaquSat5-3102-F2	AGCATCCTGAATTGGGAAAGACAG
TaquSat5-3102-R2	GAGCCTCTGTTTTTGTGCCAGAC
TaquSat6-84-F	CATCAGAGAATTCAYACTGGAG
TaquSat6-84-R	AAARGCTTTCCCACAYTCTTCAC
TaquSat7-60	CGATCACAAGCCTCAACTAGTGGTACCTG
TaquSat8-45	AGACCATCCACCAGTCTCATCAACATCTC
TaquSat9-90-F	CAGATGAAGANAGGGAATACAGAC
TaquSat9-90-R	CACCCTGTGCTATCATTTGTCTC
TaquSat10-24	AGTCTGTGACCATGATCAAGGCTC
TaquSat11-71-F	CCTCAGCGCCTGCCACTGAAG
TaquSat11-71-R	GTGAGTGGCTGTAGACCCCTTG
TaquSat12-101-F	GGAAAACCACTGAATGACAGGAC
TaquSat12-101-R	GTTATTCTCAGACTGTGGAGATGTAG
TaquSat13-54	GCAGGATCAAGTGCAACGACAAAATCAGAC
TaquSat14-17	CAGAACCCCAGTGACCCCAGAAC
TaquSat15-64	GGCTGGGAAGGATGAGACCAAGCAATAG
